# Quercetin 7-rhamnoside reduces porcine epidemic diarrhea virus replication via independent pathway of viral induced reactive oxygen species

**DOI:** 10.1186/1743-422X-8-460

**Published:** 2011-10-04

**Authors:** Jae Hyoung Song, Jae Kwon Shim, Hwa Jung Choi

**Affiliations:** 1 Department of Herbal Resources, Professional Graduate School of Oriental Medicine, Wonkwang University, Iksan 570-749, South Korea; 2 Department of Secretarial Administration, Korea Nazarene University, Chungnam 331-718, South Korea; 3 Department of Clinical Pathology, Daejeon Health Sciences College, 77-3 Gayang2-Dong, Dong-Gu, Daejeon 300-711, South Korea

## Abstract

**Background:**

On the base of our previous study we were observed relevant studies on the hypothesis that the antiviral activity of quercetin 7-rhamnoside (Q7R), a flavonoid, won't relate ability of its antioxidant.

**Methods:**

We were investigated the effects of Q7R on the cytopathic effects (CPE) by CPE reduction assay. Production of DNA fragment and reactive oxygen species (ROS) induced by PEDV infection were studied using DNA fragmentation assay and flow cytometry.

**Results:**

In the course of this study it was discovered that Q7R is an extremely potent compound against PEDV. The addition of Q7R to PEDV-infected Vero cells directly reduced the formation of a visible cytopathic effect (CPE). Also, Q7R did not induce DNA fragmentation. Furthermore, ROS increased the infection of PEDV, which was strongly decreased by N-acetyl-L-cysteins (NAC). However, the increased ROS was not decreased by Q7R. Antiviral activity of antioxidants such as NAC, pyrrolidine dithiocarbamate (PDTC), and the vitamin E derivative, trolox, were hardly noticed.

**Conclusions:**

We concluded that the inhibition of PEDV production by Q7R is not simply due to a general action as an antioxidants and is highly specific, as several other antioxidants (NAC, PDTC, trolox) are inactive against PEDV infection.

## Background

Many viruses are capable of inducing cell death, leading to lysis of the infected cells [1-7]. In late stages of virus infections, morphological changes, commonly known as cytopathic effect (CPE), can be microscopically observed. Virus-induced CPE is characterized by cell rounding, shinkage, deformation of nuclei and chomatin condensation. However, early death of infected cells may limit virus replication [8]. Also, apoptosis, or programmed cell death (PCD), during the late phase of viral infection has been suggested to play an important role in virus life cycle by facilitating viral progeny release and propagation [9,10]. PCD is a process by which damaged, aged, or otherwise unwanted cells are eliminated though a series of steps that results in the destruction of their genome. The form of PCD known as apoptosis is characterized by a series of morphological changes, including nuclear condensation and fragmentation, cytoplasmic blebbing, and cell shinkage [4].

Many viruses are capable of inducing reactive oxygen species (ROS) production. Results from many studies suggest that ROS are not directly involved in the induction of apoptosis in virus-infected cells [11,12]. On the other hand, it has been demonstrated that virus infection increases the production of superoxide anion radicals from neutrophils and macrophages infiltrated into the lung of mice [13], while transgenic mice carrying over-expressed extracellular superoxide dismutase exhibited less severe lung injury after influenza virus infection [14]. These studies, therefore, postulated that the pathogenesis of virus infection involves not only the virus proliferation mediated apoptotic cell death in the infected cells, but also the direct ROS-induced cellular injury by neutrophils and macrophages infiltrated into the virus-infected organs. But, despite many studies, the events leading to the generation of ROS during viral infections are still unclear.

In this paper, we was demonstrated the effects of quercetin 7-rhamnoside (Q7R) on production of CPE, ROS and DNA fragmentation inducted by PEDV infection and also studied the relationship of antiviral and antioxidant activity between Q7R and antioxidants.

## Methods

### Chemicals

Ribavirin and sulforhodamine B (SRB) were purchased from Sigma-Aldrich (St. Louis, MO, USA). All other chemicals were a reagent grade. Q7R was isolated from aerial parts of *Houttuynia cordata *using a previously described method [15].

### Viruses, Cell lines and Reagents

Vero (an african green monkey kidney cell line; ATCC CCR-81) was kindly provided by ATCC (American Type Culture Collection, Manassas, VA, USA). PEDV CV 777 (porcine epidemic diarrhea virus) was obtained from national veterinary research & quarantine service in Korea. Vero cells were maintained in minimal essential medium (MEM) supplemented with 10% fetal bovine serum (FBS) and 0.01% antibiotic-antimycotic. Antibiotic-antimycotic, trypsin-EDTA, FBS and MEM were supplied by Gibco BRL (Grand Island, NY). The tissue culture plates were purchased from Falcon (BD Biosciences, NJ, USAs). Virus stock was stored at -70°C until use.

### Assays of antiviral activity and cytotoxicity

The antiviral activity and cytotoxicity of Q7R against viruses were determined by cytopathic effect (CPE) reduction method recently reported [15]. Also, the effect of Q7R on PEDV-induced CPE was observed by cytopathic effect (CPE) reduction method recently reported [15]. Ribavirin was used as positive, and was solublized in dimethylsulfoxide (DMSO) used as negative control.

### Measurement of ROS induced by infection of PEDV

The level of intracellular ROS was measured by the alteration of fluorescence resulting from oxidation of 2', 7'-dichlorofluorescein diacetate (DCFH-DA, Molecular Probes, Eugene, OR). DCFH-DA was dissolved in DMSO to a final concentration of 20 mM before use. For the measurement of ROS, cells were treated with Q7R and other reagents for a time period indicated in the figure legends. After washing twice with cold PBS, they were incubated with 20 μM DCFH-DA at 37°C for 15 min. DCFH-DA is a stable compound that easily diffuses in to cells and is hydrolyzed by intracellular esterase to yield a reduced, non-fluorescent compound, DCFH, which is trapped within cells. The ROS produced by cells oxidized the DCFH to highly fluorescent 2', and 7'-dichlorodihydrofluorescein (DCF). The intensity of fluorescence was recorded using a flow cytometry (Becton Dickenson), with an excitation filter of 530 nm and an emission filter 575 nm. The ROS level was calculated as a ratio of: ROS = mean intensity of exposed cells: mean intensity of unexposed cells.

### DNA fragmentation assay

Vero cells were seeded onto a 6-well culture plate at a concentration of 2 × 10^4 ^cells per well. Next day, medium was removed and the cells were washed with PBS. Then, 0.09 ml of diluted virus suspension and 0.01 ml of medium supplemented with typsin-EDTA containing an appropriate concentration of the antiviral compound were added. It was a ten-fold dilution scheme for each compound. The culture plates were incubated at 37°C in 5% CO_2 _for 2 days, the cells were lysed with lysis buffer (TE buffer;10 mM Tris-HCl, pH 8.0, 100 mM NaCl, 10 mM EDTA, 0.5% SDS] and incubated for 10 min on ice, then centrifuged at 13,000 rpm for 30 min at 4°C. Cysolic DNA was extracted by phenol: chlororform (1:1) extraction of the supernatants. DNA was treated with 0.1 mg/ml Rnase A for 30 min at 37°C. The DNA was separated by agarose gel electrophoresis, and the DNA fragmentation was visualized from the digitized image of the gel as described [12].

## Results

### The effect of Q7R on PEDV-induced CPE

**Figure 1:**
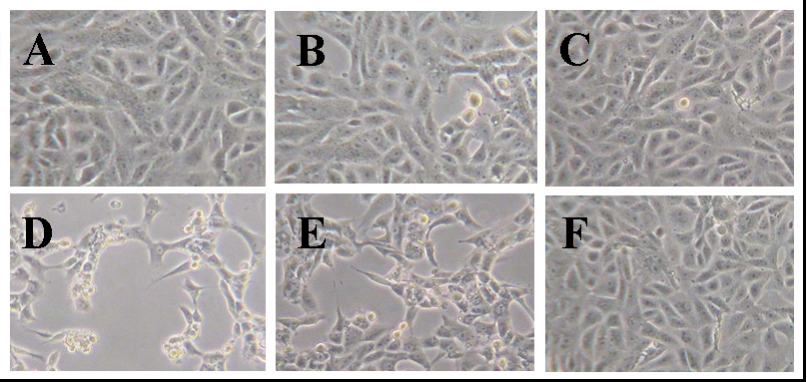
**The effects of Q7R on PEDV-induced CPE**. Culture medium 6-well tissue culture plates were removed and the cells were washed with PBS. Then, 0.09 ml of diluted virus suspension and 0.01 ml of medium supplemented with typsin-EDTA containing Q7R of 10 μg/ml were added. After incubation at 37°C in 5% CO_2 _for 2 days, the morphology of cells was investigated under microscope and a photograph taken. (A) Non-infected cells; (B) PEDV-infected cells with Q7R; (C) non-infected cells with Q7R; (D) PEDV-infected cells without Q7R; (E) PEDV-infected cells with ribavirin; (F) non-infected cells with ribavirin.

After 2 day infections of Vero cells with PEDV, Mock cells (Figure 1A) or cells treated with 10 μg/ml Q7R (Figure 1C) or ribavirin (Figure 1B) showed typical spread-out shapes and normal morphology. At this concentration, no signs of cytotoxicity of Q7R were observed. Infection with PEDV in the absence of Q7R resulted in a severe CPE (Figure 1D). Addition of Q7R on infected Vero cells inhibited the formation of a visible CPE (Figure 1F). However, the addition of ribavirin in PEDV-infected Vero cell was impossible to prevent CPE (Figure 1E). Thus, the CPE of the virus infection is prevented by the presence of Q7R.

### PEDV infection leads to ROS generation

**Figure 2:**
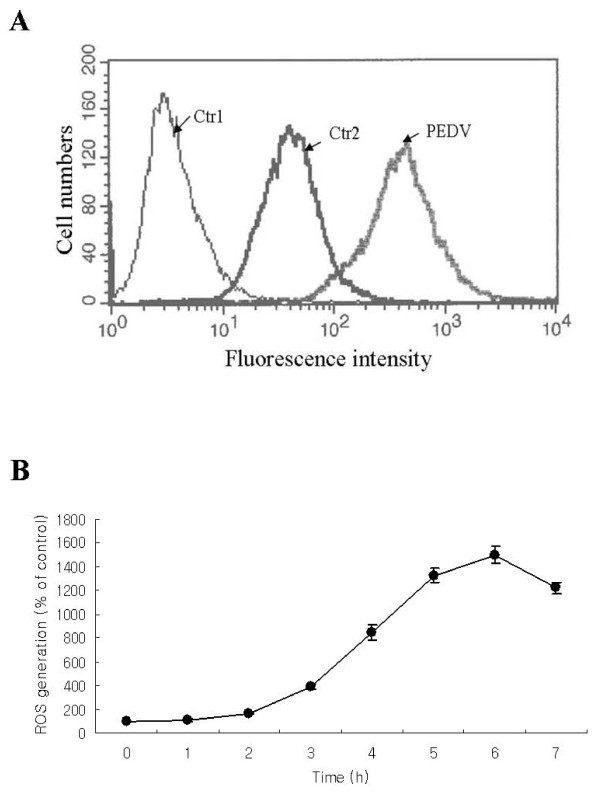
**Reactive oxygen species generation is increased during PEDV replication**. Vero cells were infected with PEDV (MOI = 10) for the indicated time. Redox-sensitive fluorescence probe DCFH-DA (20 μM) was added to the phosphate buffered-saline (pH 7.2) after harvest of Vero cells infected with PEDV. A representative flow cytometry histogram of cells infected with PEDV is shown. Time is post-PEDV infection. All data represent mean values of thee independent measurements ± S.D. Ctr1, control untreated with DCFH-DA; Ctr2, control treated with DCFH-DA; PEDV, infection with PEDV for 7 h.

To investigate the possible mechanisms of the observed antiviral effects of Q7R, we first examined whether the antioxidant property of Q7R contributed to its action. To determine the influences of PEDV replication on intracellular ROS level, Vero cells were infected with PEDV for various periods of time, and a fluorescence probe, DCFH-DA, was added to the medium prior 15 min to use a flow cytometry. As shown in Figure 2A and 2B, exposure to PEDV resulted in increased ROS production which began at 2 h post-infection and peaked at 6 h (Figure 2A and 2B).

### The effect of Q7R on ROS increased by PEDV infection

**Figure 3:**
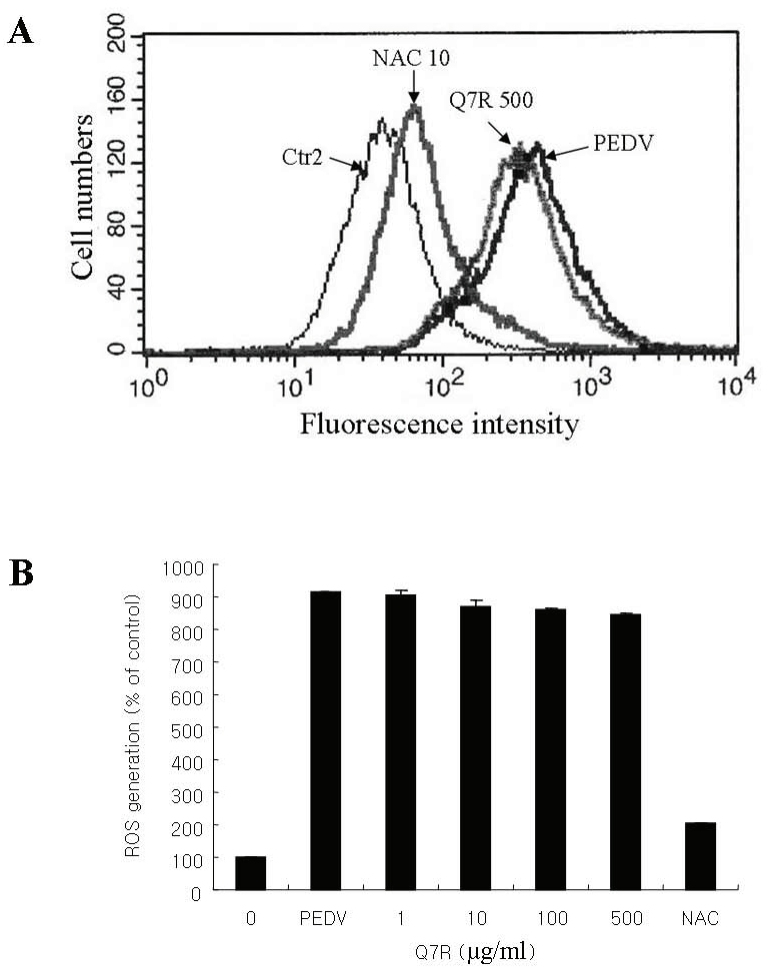
**Inhibition of PEDV replication by Q7R is independent of its antioxidant activity**. Vero cells were infected with PEDV (MOI = 10) in the presence of Q7R. Relative ROS generation in Vero cells exposed to Q7R of 1, 10, 100 and 500 μg/ml for the indicated times. NAC exposed to 10 μg/ml for the indicated times. Redox-sensitive fluorescence probe DCFH-DA (20 μM) was added to the phosphate buffered-saline (pH 7.2) after harvest of Vero cells infected with PEDV at 6 h. Representative images of ROS-induced DCF fluorescence of infected cell at 6 h post-infection are shown at a flow cytometry histogram. All data represent mean values of six independent measurements ± S.D. Ctr2, control treated with DCFH-DA; NAC 10, treated with N-acetyl L-cysteine of 10 μg/ml; PEDV, infected with PEDV for 6 h; Q7R 500, treated with Q7R of 500 μg/ml.

To study the influence of antioxidant on ROS increased by PEDV infection, Q7R and NAC were used. As shown in Figure 3A and 3B, at 3 h post-infection, PEDV infection resulted in a drastic increase of intracellular ROS, which was strongly decreased by NAC but not by Q7R, according to increase of its concentration.

### Antiviral activity of antioxidants

**Figure 4:**
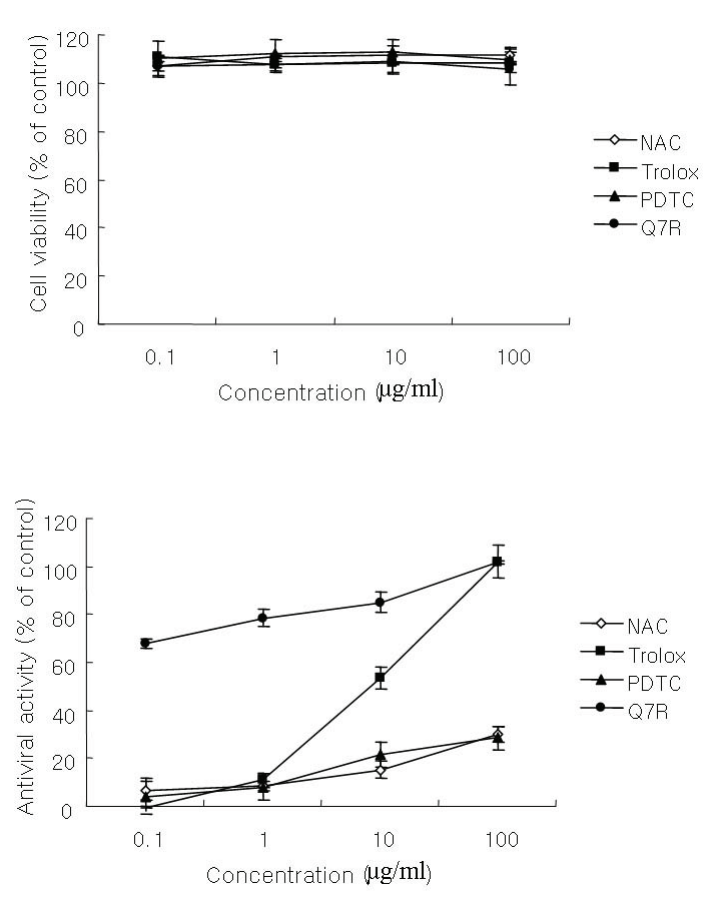
**Comparison of Q7R with several antioxidants on antiviral activity and cell viability**.

To study the influence of antioxidants on PEDV replication, the antioxidants, NAC, trolox and PDTC were used. As shown in Figure 4A and 4B, the four compounds did not exhibit cytotoxicity at different concentrations. Antiviral activity of NAC and PDTC was hardly present. However, trolox strongly exhibited antiviral activity at 100 μg/ml concentration, but decreased rapidly according to a dose dependent manner compared with Q7R.

### DNA fragmentation assay

**Figure 5:**
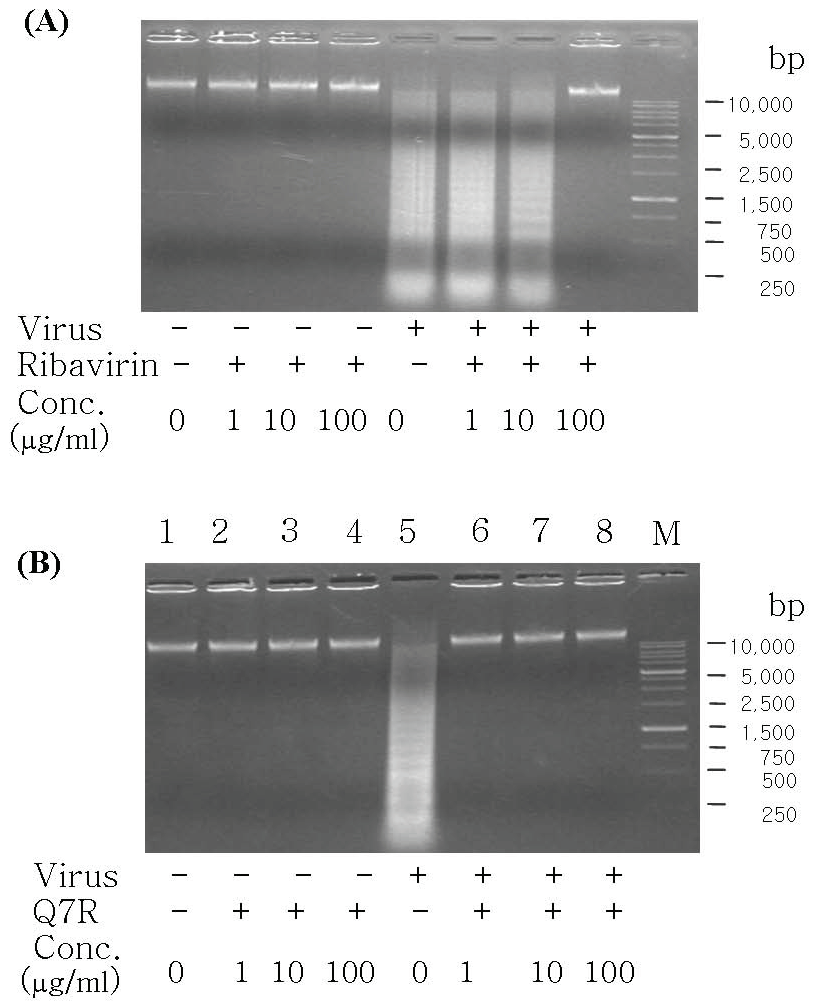
**Inhibitory effect Q7R on DNA fragmentation inducted by infection of PEDV**. (A) Vero cells were cultured for 48 h in the absence or presence of ribavirin at concentration of 1, 10 and 100 μg/ml with PEDV infection. (B) Vero cells were cultured for 48 h in the absence or presence of Q7R at concentration of 1, 10 and 100 μg/ml with PEDV infection. Profiles of agarose gel electrophoresis of DNA fractions extracted from the cells are shown. Lane M showed DNA size markers.

The effect of Q7R on the extent of DNA fragmentation resulting from PEDV infection was examined. The incubation with Q7R or ribavirin up to 100 μg/ml concentration for 48 h did not induce DNA fragmentation in mock-infected Vero cells (Figure 5A or 5B, lanes 2-4). PEDV infection induced DNA fragmentation in Vero cells 48 h after infection in the absence of compounds (Figure 5A or 5B, lanes 5). In the presence of Q7R, the DNA fragmentation was not induced in a dose-dependent manner (Figure 5B, lanes 6-8). But, DNA fragmentation was somewhat decreased when Vero cells infected with PEDV were treated with ribavirin at concentration of 1 or 10 μg/ml for 48 h. Incubation with ribavirin of 100 μg/ml did not induce DNA fragmentation.

## Discussion

Many viruses are capable of inducing cell death, leading to lysis of infected cells [1-3]. In late stages of PEDV infections, morphological changes commonly known as CPE, microscopically observed. The morphology of Vero cells after infection with PEDV was greatly decreased from that of PEDV by addition of Q7R. However, the addition of ribavirin to PEDV-infected Vero cell proved to be impossible in preventing CPE.

Viral infections such as rhinovirus, influenza virus, human immunodeficiency virus and bovine viral diarreha virus frequently result in the generation of oxidative stress in the infected cells [4-7]. The events leading to the generation of ROS during viral infections are still unclear. Also, antioxidants have been shown to have antiviral activities against a variety of unrelated viruses by alleviating the oxidative stress generated by viruses [16-20]. Mechanistically, it is believed that these viruses induce apoptosis by oxidative stress mediated via ROS. Interference with this pathway by antioxidants is believed to inhibit virus-induced apoptosis and thus inhibit efficient virus multiplication. In contrast, there are also reports indicating that under certain conditions compounds act as a pro-apoptotic drug [21-23]. Depending on the viral system analyzed, antioxidative compounds differ in their ability to reduce virus growth [7,24,25].

Flavonoids are a large class of polyphenolic compounds and Jung et al. (2003) reported that the relationship between flavonoid structure and antioxidant activity. They found that the inhibitory activities of flavonoids on total ROS are more strongly increases with the rising number of hydroxyl groups than the foavonoid glycosides on their structures [26].

Our previous study showed that quercetin 7-rhamnoside (Q7R) didn't directly interact with PEDV particles and affect the initial stage of PEDV infection by interfering with its viral mRNA production [15]. In this report, we present evidence that Q7R, but not other commonly used antioxidants, are able to protect cells from PEDV induced death. Q7R are potent agents that have been shown to be involved in a number of processes, suggesting that their antiviral effects might not be due to its antioxidant functions alone. Nevertheless, further studies are needed to verify the underlying mechanism of Q7R action in inhibiting PEDV infection.

In conclusion, Q7R is an extremely potent anti-PEDV substance which reduces PEDV growth, inhibits the CPE and DNA fragment of infected cells regardless of its antioxidant activity and then didn't directly interact with PEDV particles and affect the initial stage of PEDV infection by obstructing with its viral mRNA production. It will be interesting to further investigate the antiviral activity of the Q7R in preventing various PEDV-mediated injuries in *in vivo *pathological situations.

## Competing interests

The authors declare that they have no competing interests.

## Authors' contributions

^1^JHS performed the exam wholly. ^2^HJC conceived of the study, and participated in its design and drafted the manuscript. All authors read and approved the final manuscript.
